# Development and validation of a machine learning-based fall-related injury risk prediction model using nationwide claims database in Korean community-dwelling older population

**DOI:** 10.1186/s12877-023-04523-8

**Published:** 2023-12-11

**Authors:** Kyu-Nam Heo, Jeong Yeon Seok, Young-Mi Ah, Kwang-il Kim, Seung-Bo Lee, Ju-Yeun Lee

**Affiliations:** 1 College of Pharmacy and Research Institute of Pharmaceutical Sciences, Seoul National University, 1, Gwanak-Ro, Gwanak-Gu, Seoul, 08826 Republic of Korea; 2 College of Pharmacy, Yeungnam University, Gyeongsan-si, 38541 Republic of Korea; 3 Department of Internal Medicine, Seoul National University Bundang Hospital, Seongnam, 13620 Republic of Korea; 4 Department of Internal Medicine, Seoul National University College of Medicine, Seoul, 03080 Republic of Korea; 5 Department of Medical Informatics, Keimyung University School of Medicine, Dalgubeol-Daero 1095, Dalseo-Gu, Daegu, 42601 Republic of Korea

**Keywords:** Fall, Fall-related injury, Older adults, Machine-learning, Prediction model, Claims data

## Abstract

**Background:**

Falls impact over 25% of older adults annually, making fall prevention a critical public health focus. We aimed to develop and validate a machine learning-based prediction model for serious fall-related injuries (FRIs) among community-dwelling older adults, incorporating various medication factors.

**Methods:**

Utilizing annual national patient sample data, we segmented outpatient older adults without FRIs in the preceding three months into development and validation cohorts based on data from 2018 and 2019, respectively. The outcome of interest was serious FRIs, which we defined operationally as incidents necessitating an emergency department visit or hospital admission, identified by the diagnostic codes of injuries that are likely associated with falls. We developed four machine-learning models (light gradient boosting machine, Catboost, eXtreme Gradient Boosting, and Random forest), along with a logistic regression model as a reference.

**Results:**

In both cohorts, FRIs leading to hospitalization/emergency department visits occurred in approximately 2% of patients. After selecting features from initial set of 187, we retained 26, with 15 of them being medication-related. Catboost emerged as the top model, with area under the receiver operating characteristic of 0.700, along with sensitivity and specificity rates around 65%. The high-risk group showed more than threefold greater risk of FRIs than the low-risk group, and model interpretations aligned with clinical intuition.

**Conclusion:**

We developed and validated an explainable machine-learning model for predicting serious FRIs in community-dwelling older adults. With prospective validation, this model could facilitate targeted fall prevention strategies in primary care or community-pharmacy settings.

**Supplementary Information:**

The online version contains supplementary material available at 10.1186/s12877-023-04523-8.

## Introduction

Falls in older adults are a major public health problem [1]. They can occur in any age, but the incidence and severity of fall and fall-related injuries increase with age [2, 3]. More than one out of four older adults fall annually, 10% of older adults reported an injury from a fall [2], and falls are a leading cause of death from unintentional injury [4]. Problems caused by falls are not limited to physical problem. Traumatic falls can develop into fear of falls, which subsequently leads to complications, such as restriction of activities, anxiety, and depression, negatively affecting an individual’s quality of life [5]. Moreover, fear of falls is an independent risk factor for falls among older adults [6]. As the population is aging and the burden of falls is expected to increase, establishing effective fall prevention strategies is an urgent task in the healthcare system.

Fall-risk-increasing drugs (FRIDs) include antihypertensives, diuretics, analgesics, antidepressants, antipsychotics, and hypnotics [7, 8]. Polypharmacy and FRIDs, especially psychotropic drugs, are the drug-related risk factors for falls. Lotta et al. performed an adjusted meta-analysis of 248 studies and found that antipsychotics, benzodiazepines, and antidepressants increased the odds of falls by 1.54 (95% confidence interval [CI], 1.28–1.85), 1.57 (95% CI, 1.43–1.74), and 1.42 (95% CI, 1.22–1.65), respectively [9]. Moreover, Dalwhani et al. observed increased incidence rates of falls with 20% and 50% higher in patients receiving > 4 and > 10 drugs, respectively [10].

Interventions for medications that increase/decrease fall risk are some of the most effective fall prevention strategies [11]. The American Geriatrics Society and British Geriatrics Society guidelines on fall prevention recommend withdrawal or minimization of psychoactive medications and total number of medications [11]. A previous study that performed a meta-analysis on 14 randomized controlled trials to evaluate the effects of medication review on fall prevention in community-dwelling older adults revealed that adjusting medications that were associated with falls could decrease the risk of falls, although the risk difference was modest [12]. However, according to a recent randomized clinical trial, which aimed to determine the clinical efficacy of a multifactorial intervention in a primary care setting on fall prevention, the multifactorial intervention did not result in a significantly lower rate of serious falls than enhanced user care among older adults with risk factors for falls [13]. The fact that there were little interventions on FRIDs could be the reason why the multifactorial intervention was not effective. In this study, only 29% of the participants who were taking FRIDs agreed to address medication-related risk factors and were the least prioritized risk factor.

Several tools have been validated and widely used to predict and prevent falls in the primary care setting [14–20]. The guidelines on fall prevention recommend that these tools be used to assess the risk of falling, but there is no clear guide to which tools to use [21]. Recently, with the development of technology, predictive models using advanced analytics are being actively developed, but only a limited number of studies have used machine learning to predict falls in community-dwelling older adults [22–26]. Ikeda et al. developed a prediction model with eXtreme Gradient Boosting (XGBoost) algorithm using prospectively collected survey data [22]. Makino et al. also used survey data and developed a decision tree model [23]. Ye et al. fitted five different machine learning algorithms using electronic health record data with features comprising demographics, clinical utilization, disease diagnosis, and medication prescriptions [24]. Mishra et al. also used electronic health record data to fit four different machine learning algorithms with features comprising gait measurements, demographics, and several geriatric assessment scores [25]. Engels et al. fitted an ensemble machine learning model using administrative claims database with features comprising demographics, fall history, and medication use [26]. However, previous studies have several key limitations, such as not considering medications as risk factors (or including only polypharmacy as a risk factor) [22, 23, 25], not attempting to interpret the model [26] or interpret the model solely based on the result of univariate odds ratio [24], and having small sample sizes that limited generalizability to the entire population [25]. In addition, no study has attempted to validate the machine learning algorithms on external cohorts with different time periods.

We aimed to develop and externally validate an interpretable machine learning-based fall-related injury (FRI) prediction model using claims database especially focusing on extensive range of medications. Using this tool, we expect to identify patients at high risk for FRIs and to provide medication intervention strategies for fall prevention in older adults.

## Methods

### Data source

This retrospective cohort study was conducted with the data obtained from the Korean Health Insurance Review and Assessment Service – Aged Patient Sample (HIRA-APS) databases sampled annually for the year 2018 and 2019. In Korea, the national health insurance system provides coverage for 98% of the populations, and the HIRA database contains claims data for over 90% of the population assuring generalizability of analysis [27]. The HIRA-APS dataset is a 10% stratified random sample of claims data for patients aged > 65 years and contains comprehensive information on patient demographics, disease diagnoses based on the International Statistical Classification of diseases Tenth Revision, procedures, and prescriptions details.

### Cohort description

From July to September of each year, we identified older adults in the outpatient setting and set the cohort entry date as the date when the patient received a prescription for medications lasting > 30 days in ambulatory care. For robust operational definition, following criteria were applied to register patients: (a) patients were excluded if there was no ambulatory prescription prior to 6 months from cohort entry date; (b) patients were excluded if they had been hospitalized for > 150 days out of 6 months before the cohort entry date, (c) patients were excluded if evidence of recent FRI (diagnostic code of FRIs at any position) presented 3 months prior to entry date, and (d) patients who died without observation of any FRIs within 3 months from entry date were excluded (Supplementary Figure S1). To note, exclusion criteria (c) was specifically applied to reduce the misclassification of individuals undergoing treatment for previous FRI as incident fall, in line with methodologies from prior studies [28].

### Outcome and follow-up

The outcome of interest was the incidence of serious FRI. We operationally defined outcome as presence of emergency department (ED) visit or admission with primary or first secondary diagnostic code of non-pathological fracture of the skull, face, cervical region, clavicle, thorax, lumbar region, humerus, forearm, pelvis, hip fibula, tibia, and ankle or brain injury or dislocation of the lumbar region, pelvis, hip, knee, shoulder, elbow, cervical region, thorax, or jaw (Supplementary Table S1). Although the operational definition was determined with reference to previous studies [29, 30], external codes indicative of FRIs could not be utilized because they were masked from the data for privacy and security reasons. Patients were followed up from entry date until either of the following, whichever occurred earlier: (a) occurrence of FRI, (b) death, and (c) study end date (the last day of each year).

### Candidate features

We collected 187 candidate features previously reported as risk factors for falls and were captured at claims database (Supplementary Table S2) [9, 24, 26, 31–37]. They included demographics (age, sex, insurance status), healthcare utilization pattern, prior FRIs, specific diagnoses, exposure to FRIDs and other medications that increase/decrease the incidence of FRIs, drug–drug interactions, and drug–disease interactions. Demographics, medication, drug–drug interactions, and drug–disease interactions were assessed at the time of entry date (for medication exposure, fill date and days supplied were considered), whereas other features were assessed in the 6-month window before the entry date.

### Machine learning algorithms and model development

In this study, we divided the patients from the 2018 database into a development cohort and those from 2019 into a validation cohort. To enhance both the accountability and the clarity of our prediction model, we selected four explainable machine learning algorithms: Random forest (RF), XGBoost, Light Gradient Boosting Machine (LightGBM), and CatBoost. Our goal was to construct a model that was not only accurate but also comprehensible in its predictive processes. Traditionally, while these decision tree ensemble models have been highly accurate, their ‘black box’ approach often hampered practical application due to a lack of interpretability. Recent advancement in interpretative frameworks have, however, considerably expanded their applicability in healthcare decision-making [38]. For comparative analysis, we included a logistic regression model as a reference.

In the initial phase with the development cohort, association among features was analyzed using Spearman’s rank correlation, and the features were filtered to ensure that there were no features with a coefficient exceeding 0.9, to avoid multicollinearity. Next, the optimal set of features was explored via sequential backward floating selection [39]. To streamline the feature selection process, we implemented two strategies: initially, we downsized the development cohort through one-sided selection to achieve a 1:4 ratio of fallers (minority class) to non-fallers (majority class). Subsequently, we employed the LightGBM model for feature selection, capitalizing on its efficiency and rapid processing capacity for large datasets. Fivefold cross-validated area under the receiver operating characteristic (AUROC) curves was used as the metrics for model assessment, and 1-standard error rule was applied to select the most parsimonious model [40]. Using this approach, we were able to eliminate features with low importance while maintaining the performance and increasing the interpretability of the model. After the selection of the final list of features, hyperparameter was tuned with the entire development cohort for each machine learning model using Optuna [41]. In total, 1,000 trials were conducted, and hyperparameter combinations with the highest AUROC were saved for each model. During this process, again, fivefold cross-validation was used. Explored parameter fields and selected parameters are shown in Supplementary Table S3.

### Performance measures

All prediction performance was measured at the validation cohort. To assess discrimination performance, we measured the AUROC at 3 months. The cutoff point was determined by maximizing the Youden index [42]. We reported other metrics, including sensitivity, specificity, positive predictive value (PPV), and negative predictive value (NPV), at the cutoff point determined using the Youden index. In addition, cumulative incidence plot was depicted to graphically show the difference of fall risk stratified by the model’s cutoff point. Calibration was visually measured by depicting calibration plot. Finally, we used SHapley Additive exPlanations (SHAP) for model interpretation [38].

### Statistical analyses

For comparison of patient characteristics, we used percentage or mean (standard deviation). The χ^2^ or Fisher’s exact test was applied to compare categorical variables between groups, whereas t-tests were used to compare continuous variables between groups. The Spearman rank correlation was used to analyze the correlation among the features. To investigate the association between occurrence of FRI and each feature, logistic regression was performed. The DeLong test was conducted to compare the difference of AUROC. Statistical significance was defined as *p*-value < 0.05. All analyses were performed using SAS version 9.4 and Python version 3.9.7.

## Results

### Characteristics of the development and validation cohorts

Out of a total of 1,475,818 older patients, 520,603 from 2018 dataset were registered in development cohort and 552,731 from 2019 were registered in validation cohort (Supplementary Figure S2). Although most variables showed statistically significant difference owing to large sample size, patient characteristics in the development and validation cohorts were similar; FRIs leading to hospitalization/ED visit were observed in 1.8% and 1.7% of the patients in the development and validation cohorts, respectively. Approximately 40% of the patients were male, and 6% had fall history and were taking seven medications per average (Table 1).

**Table 1:** Baseline characteristics of the study participants in the development and validation cohorts

**Variables, N (%)**	Development cohort (*N* = 520,603)	Validation cohort (*N* = 552,731)	*p*-value
**Fall event**	9,127 (1.8)	9,664 (1.7)	0.851
**Age group**			
65 ~ 69	160,630 (30.9)	167,732 (30.3)	< 0.001
70 ~ 74	132,592 (25.5)	141,300 (25.6)	
≥ 75	227,381 (43.7)	243,699 (44.1)	
**Male**	215,194 (41.3)	229,810 (41.6)	0.011
**Insurance: Medical aid**	37,696 (7.2)	39,244 (7.1)	0.005
**Comorbid disease**			
Prior fall-related injury	30,189 (5.8)	33,244 (6.0)	< 0.001
Hypertension	384,631 (73.9)	405,190 (73.3)	< 0.001
Chronic heart failure	45,007 (8.7)	48,653 (8.8)	0.004
Diabetes mellitus	191,089 (36.7)	207,143 (37.5)	< 0.001
Dyslipidemia	310,685 (59.7)	342,403 (61.9)	< 0.001
Ischemic heart disease	82,778 (15.9)	87,353 (15.8)	0.171
Cerebrovascular disease	72,525 (13.9)	77,448 (14.0)	0.227
Arrhythmia	31,515 (6.1)	24,745 (4.5)	< 0.001
Dorsopathy	270,476 (52.0)	291,260 (52.7)	< 0.001
Osteoporosis	113,345 (21.8)	126,037 (22.8)	< 0.001
Osteoarthritis	218,801 (42.0)	236,722 (42.8)	< 0.001
Parkinsonism	11,866 (2.3)	12,485 (2.3)	0.476
Urinary incontinence	13,273 (2.5)	14,654 (2.7)	< 0.001
**CCI score, mean ± SD**	1.79 ± 1.66	1.70 ± 1.63	< 0.001
0 ~ 2	378,273 (72.7)	412,071 (74.6)	< 0.001
3 ~ 4	107,478 (20.6)	107,150 (19.4)	
≥ 5	34,582 (6.7)	33,510 (6.1)	
**Concurrent medication**			
CCBs	235,483 (45.2)	249,311 (45.1)	0.198
ACEi/ARBs	257,955 (49.5)	276,238 (50.0)	< 0.001
Beta blockers	80,931 (15.5)	84,854 (15.4)	0.006
Loop diuretics	23,168 (4.5)	24,664 (4.5)	0.763
Sulfonylureas	59,915 (11.5)	61,565 (11.1)	< 0.001
Insulin	12,476 (2.4)	13,772 (2.5)	0.001
Corticosteroids	20,984 (4.0)	23,009 (4.2)	< 0.001
NSAIDs	111,782 (21.5)	121,863 (22.0)	< 0.001
Warfarin	4186 (0.8)	3664 (0.7)	< 0.001
DOACs	11,765 (2.3)	14,600 (2.7)	< 0.001
Opioids (excluding tramadol)	6026 (1.2)	6496 (1.2)	0.392
Tramadol	44,893 (8.6)	49,245 (8.9)	< 0.001
Benzodiazepines	61,596 (11.8)	64,304 (11.6)	0.002
Zolpidem	16,276 (3.1)	17,506 (3.2)	0.227
Gabapentinoids	23,259 (4.5)	27,122 (4.9)	< 0.001
TCAs	16,121 (3.1)	16,615 (3.0)	0.006
SSRIs	18,658 (3.6)	21,714 (3.9)	< 0.001
Antipsychotics	14,180 (2.7)	16,544 (3.0)	< 0.001
1st generation antihistamines	40,984 (7.9)	43,912 (7.9)	0.166
Acetylcholine esterase inhibitors	32,482 (6.2)	36,659 (6.6)	< 0.001
Vitamin D	22,969 (4.4)	26,268 (4.8)	< 0.001
Bisphosphonates	53,616 (10.3)	55,798 (10.1)	< 0.001
**No. of medications, mean ± SD**	7.02 ± 4.48	7.19 ± 4.58	< 0.001
0 ~ 4	174,618 (33.5)	179,300 (32.4)	< 0.001
5 ~ 9	216,109 (41.5)	227,845 (41.2)	
≥ 10	129,876 (25.0)	145,586 (26.3)	

*CCI* Charlson comorbidity index, *SD* standard deviation, *CCB* calcium channel blocker, *ACEi* angiotensin-converting enzyme inhibitor, *ARB* angiotensin receptor blocker, *NSAIDs* non-steroidal anti-inflammatory drug, *DOAC* direct oral anticoagulant, *TCA* tricyclic antidepressant, *SSRI* selective serotonin reuptake inhibitor

### Model performance

**Fig. 1:**
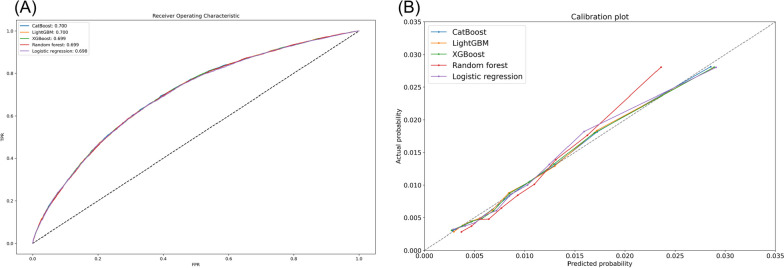
Discrimination and calibration performance of each model. **A** Receiver operating characteristic curve of each model. **B** Calibration plot of each model

After feature selection process, 26 out of the 187 candidate features were selected. The included final features were sex, age group, insurance status, number of admission or ED visit, seven comorbidities (e.g., prior FRI, dorsopathy, hyperlipidemia), 13 medication factors (e.g., number of medications, number of central nervous system [CNS] depressants, bisphosphonate, steroid), and two drug–disease interactions (e.g., CNS depressant use in patients with a history of fracture). The final list of features and their association with future fall can be found in Supplementary Table S4. The AUROC of each model is summarized in Fig. 1A. All machine learning-based models showed higher performance than logistic regression. However, the difference in performance among all five models was negligible (AUROC, 0.700, 0.700, 0.699, 0.699, and 0.698 for CatBoost, LightGBM, XGBoost, RF, and logistic regression, respectively). Calibration plot was depicted to determine if the observed and predicted probabilities were consistent (Fig. 1B). The predicted and actual probabilities of FRIs within 90 days, divided into deciles, showed concordance across all models. CatBoost was selected as our final model owing to its highest discrimination performance among the models considered.

**Fig. 2:**
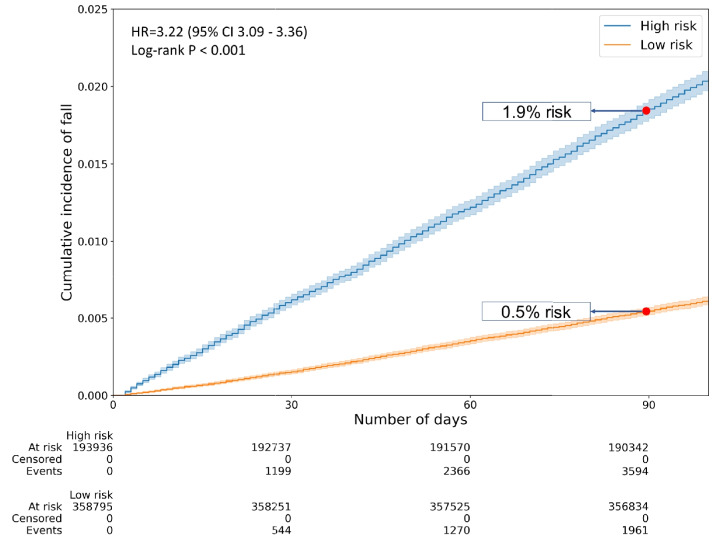
Kaplan–Meier curves for cumulative incidence of fall-related injury by risk group

Table 2 shows the performance measures of each model at the cutoff point determined using the Youden index. CatBoost showed sensitivity, specificity, PPV, and NPV of 64.7%, 65.2%, 1.9%, and 99.5%, respectively. On Kaplan–Meier analysis, there was a clear distinction of curves between risk groups (only observed in CatBoost) (Fig. 2), with the high-risk group showing more than three times higher risk of FRIs than the low-risk group (hazard ratio, 3.22; 95% CI, 3.09–3.36).

**Table 2:** Performance comparison of each model on validation cohort

Models	No. (%) of high-risk patients	No. of FRI^a^	Accuracy	Sensitivity	Specificity	PPV	NPV	AUROC	*p*-value^b^
Catboost	189,445 (34.3)	3543	0.652	0.647	0.652	0.019	0.995	0.700	< 0.001
LightGBM	202,745 (36.7)	3679	0.636	0.662	0.636	0.018	0.994	0.700	0.004
XGBoost	193,956 (35.1)	3587	0.650	0.648	0.650	0.018	0.995	0.699	0.005
Random Forest	175,115 (31.2)	3383	0.660	0.637	0.660	0.019	0.994	0.699	0.380
Logistic regression	219,624 (39.7)	3839	0.603	0.691	0.602	0.017	0.995	0.698	-

*FRIs* fall-related injury, *PPV* positive predictive value, *NPV* negative predictive value, *AUROC* area under the receiver operating characteristic curve

^a^Counted based on fall occurring within 3 months from the entry date. The total number of patients who experienced fall within 3 months was 5555

^b^*P*-value for comparison of area under the receiver operating characteristic curve with logistic regression

### Model interpretation

**Fig. 3:**
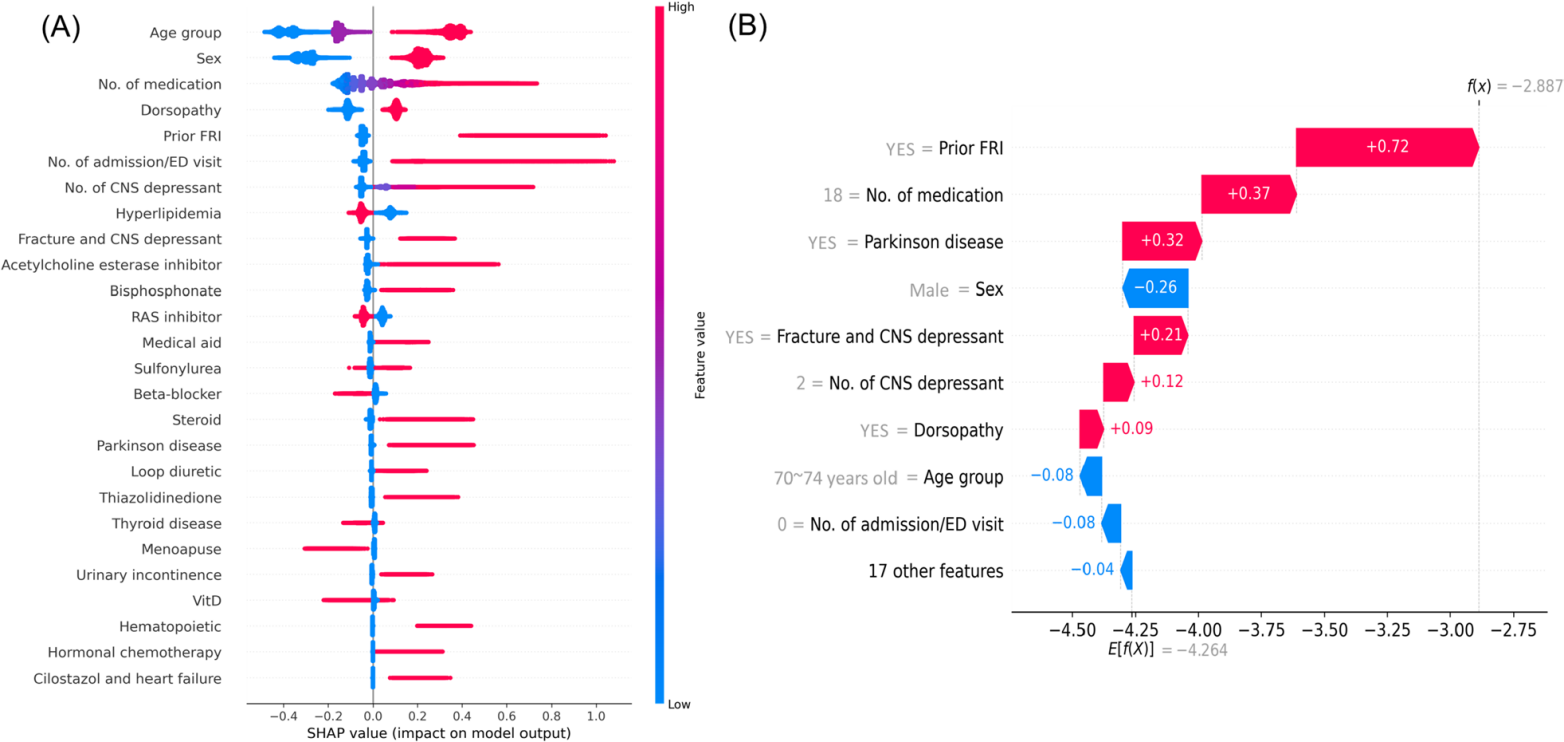
Interpretation of the model output. FRI, fall-related injury; CNS, central nervous system; ED, emergency department. **A** SHapley Additive exPlanations (SHAP) summary plot. The color represents the value of each feature, with red representing higher values and blue representing lower values. The SHAP value on the x-axis explains the direction and degree of the model’s prediction, where large positive values contribute to the prediction that a patient will experience fall-related injury, large negative values contribute to the prediction that a patient will not experience fall-related injury, and values close to zero contribute little to the prediction. **B** SHAP waterfall plot. Patient level prediction is depicted. Similarly, the SHAP value on the x-axis explains the direction and degree of the model’s prediction, where large positive values contribute to the prediction that a patient will experience fall-related injury, large negative values contribute to the prediction that a patient will not experience fall-related injury, and values close to zero contribute little to the prediction

The SHAP summary plot for CatBoost is presented in Fig. 3A, while those for other models (LightGBM, XGBoost, and RF) can be found in Supplementary Figure S3. The plot summarized the importance of features and their effects on prediction at once, with each point presenting the individual patient’s feature values and their effects on the model. The top 10 important features identified in the model were age group, sex, number of medications, dorsopathies, prior FRI, number of admission or ED visit, number of CNS depressants, hyperlipidemia, CNS depressant use with prior fracture, and exposure to acetylcholine esterase inhibitor. The model was applied to an individual patient with FRI and depicted using a SHAP waterfall plot (Fig. 3B, Figure S3). The plot represents how the prediction is made in individual patient level. Again, features were sorted in the descending order of effects on model output and also depicted their directions on prediction. Prior FRI, exposure to 18 medications, Parkinson disease, CNS depressant use with prior fracture, and exposure to two distinct CNS depressants pushed model to predict a patient will suffer from FRI, whereas male sex, absence of admission or ED visit history, and age 70–74 years pushed the model to predict a patient will unlikely to experience FRI.

## Discussion

This study developed and validated a FRI prediction model in the community-dwelling older adults using claims database. Our best performing model showed a fair ability to discriminate individuals who experienced FRI and those who did not [43] (AUROC, 0.70). By focusing on 35.1% of the patients, we could capture almost two-thirds of FRIs. Contrary to expectations, the model using machine learning algorithm only showed a slight improvement in performance compared with logistic regression. This trend is also demonstrated in a prior study conducted to predict falls with administrative claims database that shared similar characteristic of features with our study [26]. The model’s selected features and interpretation aligned well with clinical intuition. Specifically, our model predicted older adults, female sex, and prior FRI; the higher the number of CNS depressants and the higher the number of total medications, the more likely that an individual will experience FRI [34]. Our model identified dorsopathy as an important risk factor for FRIs, which is also consistent with the results of prior studies that have revealed back pain as an independent risk factor for fall [44]. Contrary to our intuition, the use of certain antihypertensives was associated with a lower risk of FRI in our study. Although the mechanism is not totally understood, similar trend has been observed in other studies [32, 45]. A meta-analysis conducted by de Vries et al. reported that beta-blockers showed protective effect against falls [32]. Ang et al.’s meta-analysis also demonstrated that beta-blockers and angiotensin-converting enzyme inhibitors were associated with lower risk of injurious fall [45]. In contrast, Butt et al. found that the incidence rate of falls was significantly higher within the first 14 days after the initiation for all classes of antihypertensives [46]. Taken together, these studies suggest that antihypertensives may increase the risk of falls in the initiation period, not in the maintenance period.

Additional care needs to be taken in interpreting this model. For instance, exposure to bisphosphonate seems to increase the risk of FRIs, but it would rather more reasonable to interpret it as the population has underlying condition with osteoporosis. Similarly, hyperlipidemia and menopause appear to be protective against falls, possibly due to the increase in bone density resulting from the use of statins or hormone replacement therapy rather than the disease itself [47, 48]. Hence, when interpreting the output of the model (which is entirely dependent on the user), it is necessary to determine whether the result is due to the influence of the medication or whether it is simply a result of the modeling process.

Our study has some limitations. First, our model’s performance was not optimal, with an AUROC of 0.70, compared with other previous machine learning-based fall prediction models (AUROC range, 0.70–0.88) [22–26]. This is possibly because physical examination results, such as gait and muscle strength, and laboratory values, such as bone mineral density, which are potentially key features for predicting FRIs, cannot be obtained from claims database. Second, owing to the nature of claims database, it is not known whether the individual actually took the prescribed medications. Third, while the diagnostic codes utilized for identifying FRIs are informed by prior studies [29, 30], they may not be exclusively attributable to falls. The possibility that the injuries could be from other causes, such as vehicular accidents, cannot be entirely excluded, given that the external cause of injury codes were obscured in our dataset. However, substantial evidence suggests that a significant proportion of non-intentional injuries among older adults are caused by falls. For instance, from 2016 to 2020, fall accounted for 57% of fatal unintentional injuries and 65% of non-fatal unintentional injuries in this demographic [49]. This data substantiates the likelihood that any misclassification bias in our study would not substantially affect the validity of our findings. Fourth, given the nature of HIRA-APS dataset, it is worth noting that the data are sampled annually, and there is possibility that the same patients may be included in both 2018 sample database for model development and 2019 sample database for validation. However, due to the anonymized nature of the data, we were unable to identify duplicate patients. Nevertheless, we believe that this should not significantly impact the results.

Despite these potential limitations, our prediction model is still valuable in that it was derived from a nationally representative dataset of adult population, making it more generalizable than models based on data from a single institution. Moreover, the focus on FRIs resulting in admission or ED visit as a primary outcome underscores the clinical significance of this study and may contribute to the development of fall prevention programs that improve patient outcomes. Utilizing a claims database, our model benefits from automated data acquisition, which facilitates the identification of populations at high-risk for FRIs without additional assessment.

Our model was designed with the intention of serving as a national surveillance tool for monitoring fall-related injuries in South Korea, where the Health Insurance Review and Assessment Service (HIRA) operates a Drug Utilization Review (DUR) system. This system is instrumental in providing real-time alerts to healthcare providers about critical issues like contraindicated drug interactions, redundant prescribing, age-related contraindications, and excessive dosage [50]. Given that our model is constructed exclusively from claims data, it is conceivable that HIRA could integrate our predictive model into the DUR system to enhance its functionality. Such an advancement would allow for the automatic and real-time processing of data to pinpoint high-risk individuals, thus facilitating proactive education and timely interventions for fall-related injuries, greatly contributing to patient safety and care. Furthermore, our study stands out as the only available prediction model for FRIs in community-dwelling older adults that has been evaluated in an external validation cohort with different time periods, whereas previous studies only underwent internal validation using the random split-sample method and cross-validation.

## Conclusions

We developed and externally validated a novel explainable machine learning-based FRI prediction model using national sample claims database. We found that applying machine learning approach to predict FRIs in older adult is feasible. Although the performance is not optimal, simple and ready-to-use claims data-driven model can be utilized in routine primary care practice or community pharmacy for targeted intervention. Further prospective study is required to evaluate and validate the usefulness of the model in the clinical field.

### Supplementary Information

**Additional file 1: Figure S1.** Graphical depiction of entry date, assessment window and follow-up. **Figure S2.** Patient selection flow. **Figure S3.** SHAP summary plot for LightGBM, XGBoost, and Random Forest. **Table S1.** Diagnostic codes to identify fall-related injuries. **Table S2.** Summary of candidate features (*n*=187). **Table S3.** Explored parameter fields and selected parameters. **Table S4.** Association between selected features and fall-related injury.

## Data Availability

The datasets used in the study can be accessed from the Health Insurance Review and Assessment service, but their use is limited due to licensing and not intended for public release. However, data will be shared on reasonable request to the corresponding author with the permission of the Health Insurance Review and Assessment service.

## Abbreviations

FRIDs: Fall-risk-increasing drugs
FRI: Fall-related injury
CI: Confidence interval
XGBoost: Extreme gradient boosting
HIRA: Health insurance review and assessment service
APS: Aged patient sample
ED: Emergency department
RF: Random forest
LightGBM: Light gradient boosting machine
AUROC: Area under the receiver operating characteristic
PPV: Positive predictive value
NPV: Negative predictive value
SHAP: SHapley Additive exPlanations
CNS: Central nervous system
