# Supplementary material for: Development and validation of a machine learning-based fall-related injury risk prediction model using nationwide claims database in Korean community-dwelling older population
Source: BMC Geriatr. 2023 Dec 11;23:830. doi: 10.1186/s12877-023-04523-8 (PMC10712099; doi:10.1186/s12877-023-04523-8)
Supplement: Supplementary file 1 — Additional file 1: Figure S1. Graphical depiction of entry date, assessment window and follow-up. Figure S2. Patient selection flow. Figure S3. SHAP summary plot for LightGBM, XGBoost, and Random Forest. Table S1. Diagnostic codes to identify fall-related injuries. Table S2. Summary of candidate features (n=187). Table S3. Explored parameter fields and selected parameters. Table S4. Association between selected features and fall-related injury. [file 12877_2023_4523_MOESM1_ESM.pdf]

Supplementary material

Title: Development and validation of a machine learning-based fall-related injury risk prediction model using nationwide claims database in Korean community-dwelling older population

- Figure S1. Graphical depiction of entry date, assessment window and follow-up
- Figure S2. Patient selection flow
- Figure S3. SHAP summary plot for LightGBM, XGBoost, and Random Forest)
- Table S1. Diagnostic codes to identify fall-related injuries
- Table S2. Summary of candidate features (n=187)
- Table S3. Explored parameter fields and selected parameters
- Table S4. Association between selected features and fall-related injury

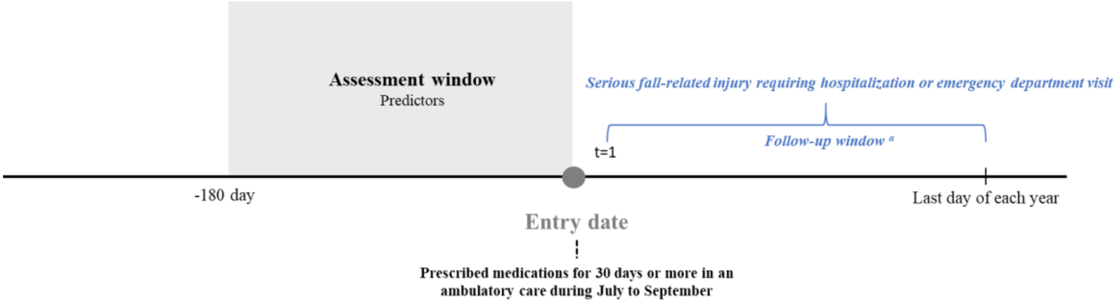

Figure S1. Graphical depiction of entry date, assessment window, and follow-up

[a] Earliest of: fall-related injury, death, the last day of the each year

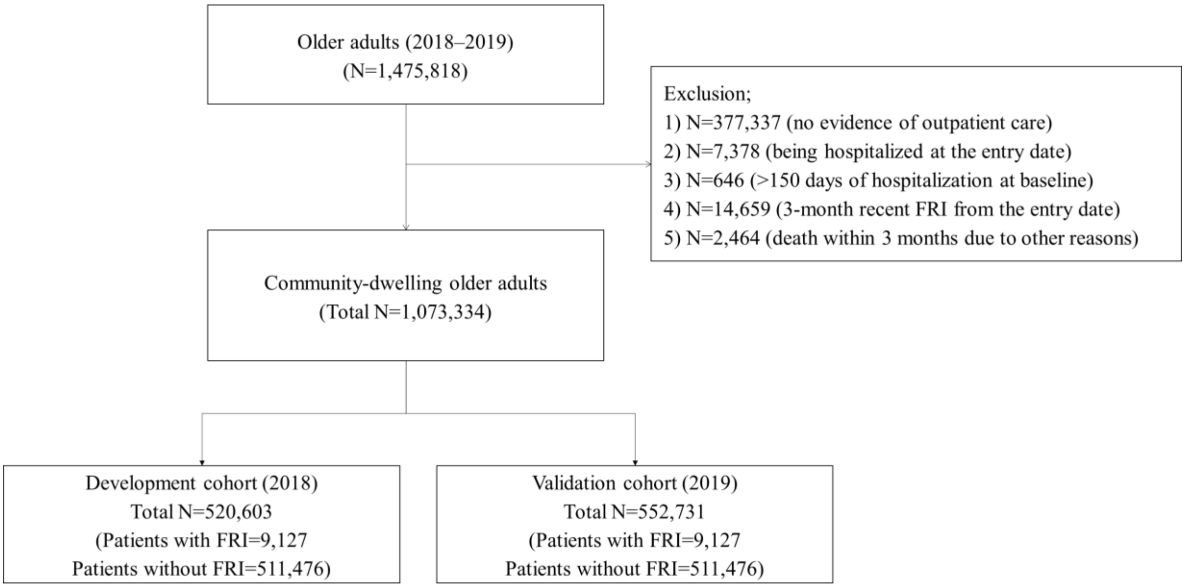

Figure S2. Patient selection flow

FRI, fall-related injury

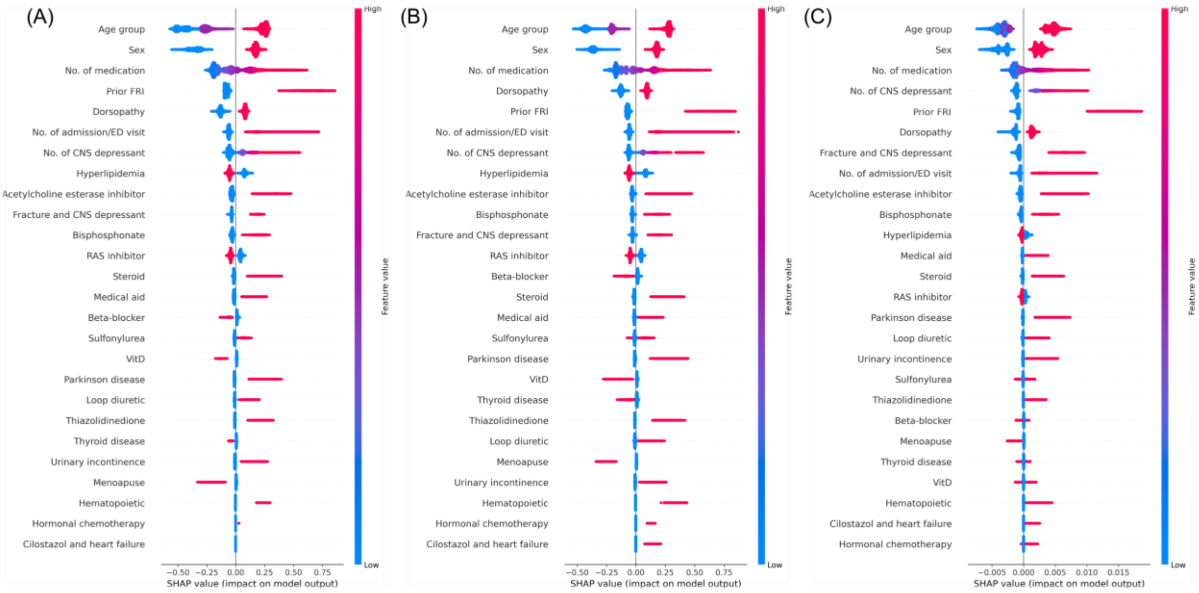

Figure S3. SHAP summary plot for LightGBM, XGBoost and Random Forest

FRI, fall-related injury; CNS, central nervous system; ED, emergency department
(A): LightGBM
(B): XGBoost
(C): Random Forest

Table S1. Diagnostic codes to identify fall-related injuries

Falls-and fall-related injury	ICD-10 code
Fall	W00~W19 *
Fracture	S02, S12, S22, S32, S42, S52, S620, S621, S72, S82, S920, T02 *, T08 *, T10 *, T12 *, T142 *
Head injury	S06, S07
Dislocation	S131, S132, S133, S231, S232, S331, S332, S333, S430, S431, S432, S433, S530, S531, S730, S830, S831

* The diagnostic codes were masked in the raw data. ICD-10, International Classification of Diseases, Tenth Revision;

Table S2. Summary of candidate features (n=186)

Demographics	Healthcare utilization	Disease	Medication	Drug-Drug interaction	Drug-disease interaction
• Sex<br>• Age group<br>• Medical aid	• Baseline hospitalization period<br>• No. of admission or ED visit<br>• No. of admission<br>• No. of ED visit	• CCI score<br>• Prior fall-related injury<br>• Pathologic fracture<br>• Ischemic heart disease<br>• Myocardial infarction<br>• Chronic heart failure<br>• Cerebrovascular disease<br>• Ischemic stroke<br>• Intracranial hemorrhage<br>• Peripheral vascular disease<br>• Thromboembolism<br>• Hypertension<br>• Arrhythmia<br>• Atrial fibrillation<br>• Bradycardia, atrioventricular block<br>• Syncope, hypotension<br>• Diabetes mellitus<br>• Type 1 Diabetes mellitus<br>• Type 2 Diabetes mellitus<br>• Hyperlipidemia<br>• Arthropathy<br>• Dorsopathy<br>• Soft tissue disease<br>• Ankylosing spondylitis<br>• Rheumatic arthritis<br>• Osteoarthritis<br>• Osteoporosis<br>• Vertigo<br>• Dizziness<br>• Renal disease<br>• Liver disease<br>• Cancer<br>• Parkinson disease<br>• Anemia<br>• Hyponatremia<br>• Hypokalemia<br>• Paralysis<br>• Glaucoma<br>• Gout<br>• Menopause<br>• Thyroid disease<br>• Hyperthyroidism<br>• Hypothyroidism<br>• Major bleeding<br>• Asthma<br>• COPD<br>• BPH<br>• Urinary retention<br>• Urinary incontinence<br>• Peptic ulcer disease<br>• Insomnia<br>• Obesity	• No. of medication<br>• No. of CNS depressants<br>• No. of fall-risk increasing drugs<br>• Opioid<br>• No. of opioid<br>• Tramadol<br>• Weak opioid<br>• Strong opioid<br>• Benzodiazepines<br>• No. of benzodiazepines<br>• Benzodiazepines (short acting)<br>• Benzodiazepines (long acting)<br>• Zolpidem<br>• Gabapentinoids<br>• Antiepileptics<br>• No. of antiepileptis<br>• Antipsychotics<br>• Typical antipsychotics<br>• Atypical antipsychotics<br>• Lithium<br>• Antidepressants<br>• No, of antidepressants<br>• TCA<br>• SSRI<br>• SNRI<br>• Antihypertensives<br>• No. of antihypertensives<br>• Vasodilators<br>• Diuretics<br>• No. of diuretics<br>• Loop diuretics<br>• Beta blockers<br>• CCB<br>• DHP CCB<br>• NDHP CCB<br>• ACEi/ARB<br>• Alpha blockers<br>• Antiarrhythmics<br>• Antiarrhythmics (class 1b)<br>• Antiarrhythmics (class 1c)<br>• Antiarrhythmics (class 3)<br>• digoxin<br>• Dopaminergic agents<br>• Traditional NSAIDs<br>• Cox-2 inhibitor<br>• Strong anticholinergic agents<br>• No. of strong anticholinergic agents<br>• 1st generation antihistamines<br>• Antidiabetic agents<br>• No. of antidiabetic agents<br>• Metformin<br>• Sulfonylurea<br>• SGLT2 inhibitors<br>• Thiazolidinedione<br>• DPP-4 inhibitor<br>• Meglitinide<br>• Alpha glucosidase inhibitor<br>• GLP-1 receptor agonist<br>• Insulin<br>• Insulin (short acting)<br>• Insulin (long acting)<br>• Insulin (premixed)<br>• Proton pump inhibitor<br>• Steroid<br>• Osteoporosis treatment<br>• Calcium<br>• Vitamin D<br>• Bisphosphonate<br>• Tibolone, raloxifene and bazedoxifene<br>• Anticoagulants<br>• Warfarin<br>• DOAC<br>• LMWH<br>• Antiplatelet agents<br>• No. of antiplatelet agents<br>• P2Y12 inhibitor<br>• Low dose aspirin<br>• Other antiplatelet<br>• Anti-anemic preparations (oral)<br>• Iron<br>• VitB12<br>• Folic acid<br>• Erythropoiesis-stimulating agents<br>• Acetylcholine esterase inhibitor<br>• Antispasmodic<br>• Muscle relaxant<br>• Thyroid hormones<br>• Antithyroid<br>• Inhaled steroids<br>• Chemotherapy<br>• Hormonal chemotherapy<br>• Anti-estrogens<br>• Aromatase inhibitor<br>• Antiandrogen<br>• Sex hormones<br>• Megestrol<br>• Metoclopramide<br>• Desmoporessin	• ACEi/ARB & potassium sparing diuretics<br>• Opioid & Gabapentinoids<br>• Opioid & Benzodiazepines<br>• Lithium & ACEi/ARB<br>• Theophylline & Cimetidine<br>• Theophylline & Ciprofloxacin<br>• Warfarin & Amiodarone<br>• Warfarin & NSAIDs<br>• Beta blocker & NDHP CCB<br>• Acetylcholine esterase inhibitor & Beta blockers/digoxin/NDHP CCB<br>• ≥2 strong anticholinergic agents<br>• ≥3 CNS depressants<br>• ≥2 Benzodiazepines<br>• ≥2 Traditional NSAIDs<br>• ≥2 SSRIs<br>• ≥2 loop diuretics<br>• ≥2 anticoagulants<br>• ≥2 antiplatelets	• Heart failure & NDHP CCB<br>• Heart failure & NSAIDs<br>• Heart failure & thiazolidinedione<br>• Heart failure & cilostazol<br>• Heart failure & dronedarone<br>• Syncope & Acetylcholine esterase inhibitor<br>• Fracture & CNS depressants<br>• Parkinson disease & antipsychotics<br>• Peptic ulcer disease & traditional NSAIDs<br>• CKD & traditional NSAIDs<br>• Bradycardia & beta blockers<br>• Bradycardia & acetylcholine esterase inhibitor

ED, emergency department; CCI, Charlson comorbidity index; COPD, chronic obstructive pulmonary disease; BPH, benign prostatic hyperplasia; CNS, central nervous system; TCA, tricyclic antidepressants; SSRI, selective serotonin reuptake inhibitor; SNRI , serotonin-norepinephrine reuptake inhibitor; CCB, calcium channel blocker; DHP, dihydropyridine; NDHP, non-dihydropyridine; ACEi, angiotensin converting enzyme inhibitor; ARB , angiotensin receptor blocker; NSAIDs, non-steroidal anti-inflammatory drugs; SGLT2, sodium-glucose cotransporter-2; DPP-4, dipeptidyl peptidase-4; GLP-1, glucagon-like peptide 1; DOAC, direct oral anticoagulant; LMWH, low molecular weight heparin; P2Y12; CKD, chronic kidney disease

Table S3. Explored parameter fields and selected parameters

Model	Hyperparameter	Parameter space	Selected parameters
LightGBM	boosting_type	goss	“goss”
	n_estimators	[100, 640]	580
	learning_rate	[0.001, 0.3]	0.5564
	num_leaves	[20, 500]	380
	max_depth	[3, 12]	11
	min_data_in_leaf	[50, 10000]	50
	lambda_l1	[0, 100]	10
	lambda_l2	[0, 100]	55
	min_gain_to_split	[0, 15]	5.8008
	feature_fraction	[0.2, 1]	0.5
CatBoost	depth	[3, 12]	6
	learning_rate	[0.001, 0.3]	0.0168
	iterations	[10, 1000]	760
	colsample_bylevel	[0.2, 1.0]	0.4555
	min_data_in_leaf	[100, 10000]	8224
	l2_leaf_reg	[0.0001, 100]	84.0688
	subsample	[0.2, 1.0]	0.6412
XGBoost	tree_method	Gpu_hist	Gpu_hist
	booster	Gbtree	Gbtree
	max_depth	[3, 32]	3
	learning_rate	[0.001, 0.3]	0.0365
	n_estimators	[50, 1000]	784
	min_child_weight	[1, 1000]	16
	gamma	[0.0001, 1.0]	0.0035
	alpha	[0.0001, 10.0]	0.0911
	lambda	[0.0001, 10.0]	0.0002
	colsample_bytree	[0.2, 1.0]	0.4442
	colsample_bylevel	[0.2, 1.0]	0.3681
	subsample	[0.2, 1.0]	0.6472
Random Forest	bootstrap	True	True
	n_estimators	[100, 1000]	511
	max_features	[3, 26]	3
	max_depth	[3, 50]	12
	min_samples_split	[10, 10000]	1236
	min_samples_leaf	[10, 10000]	82

Table S4. Association between selected features and fall-related injury

Risk factor	Univariable OR (95% CI)	p-value	Multivariable aOR (95% CI)	p-value
**Age group**				
65~69	Reference		Reference	
70~74	1.36 (1.28-1.46)	<.001	1.24 (1.16-1.33)	<.001
≥75	2.54 (2.40-2.68)	<.001	1.99 (1.87-2.1)	<.001
**Female**	2.00 (1.91-2.10)	<.001	1.74 (1.66-1.83)	<.001
**Medical aid**	1.57 (1.47-1.68)	<.001	1.13 (1.06-1.21)	<.001
**No. of admission/ED visit**	1.35 (1.31-1.38)	<.001	1.18 (1.14-1.21)	<.001
Prior fall-related injury	2.89 (2.72-3.06)	<.001	1.97 (1.85-2.11)	<.001
Hyperlipidemia	0.96 (0.92-1.00)	0.055	0.86 (0.82-0.9)	<.001
Dorsopathy	1.65 (1.58-1.72)	<.001	1.24 (1.18-1.3)	<.001
Parkinson disease	2.07 (1.87-2.29)	<.001	1.27 (1.15-1.42)	<.001
Menopause	0.81 (0.70-0.95)	0.009	0.74 (0.63-0.87)	<.001
Thyroid disease	1.12 (1.05-1.19)	<.001	0.94 (0.88-1)	0.054
Urinary incontinence	1.71 (1.54-1.89)	<.001	1.16 (1.05-1.29)	0.005
**Medication factors**				
No. of medication	1.08 (1.07-1.08)	<.001	1.04 (1.03-1.05)	<.001
No. of CNS depressant	1.29 (1.27-1.31)	<.001	1.06 (1.04-1.08)	<.001
Loop diuretic	1.60 (1.48-1.74)	<.001	1.13 (1.03-1.23)	0.007
Beta-blocker	1.08 (1.02-1.14)	0.009	0.91 (0.85-0.96)	<.001
ACEi/ARB	0.93 (0.89-0.97)	<.001	0.90 (0.86-0.94)	<.001
Sulfonylurea	1.09 (1.03-1.16)	0.006	1.07 (1.01-1.15)	0.036
Thiazolidinedione	1.27 (1.13-1.43)	<.001	1.32 (1.16-1.49)	<.001
Steroid	1.67 (1.54-1.82)	<.001	1.31 (1.2-1.43)	<.001
Vitamin D	1.33 (1.21-1.45)	<.001	0.82 (0.74-0.91)	<.001
Bisphosphonate	1.77 (1.67-1.87)	<.001	1.23 (1.15-1.31)	<.001
Acetylcholine esterase inhibitor	2.22 (2.09-2.37)	<.001	1.34 (1.25-1.43)	<.001
Hormonal chemotherapy	1.10 (0.84-1.43)	0.5	1.13 (0.87-1.48)	0.361
Hematopoietic drugs	1.80 (1.47-2.20)	<.001	1.40 (1.14-1.72)	0.001
**Drug-disease interaction**				
Heart failure and cilostazol	1.59 (1.24-2.04)	<.001	1.13 (0.88-1.45)	0.352
Fracture and CNS depressant	2.37 (2.25-2.50)	<.001	1.21 (1.13-1.29)	<.001

OR, odds ratio; aOR, adjusted odds ratio; CI, confidence interval; ED, emergency department; CNS, central nervous system; ACEi, angiotensin-converting enzyme inhibitor; ARB, angiotensin receptor blocker
